# Silver-Nanoparticle- and Silver-Nitrate-Induced Antioxidant Disbalance, Molecular Damage, and Histochemical Change on the Land Slug (*Lehmannia nyctelia*) Using Multibiomarkers

**DOI:** 10.3389/fphys.2022.945776

**Published:** 2022-08-01

**Authors:** Zeinab Bakr, Shimaa Mohamed Said, Wafaa A. Mohammad, Gehad N. Aboulnasr, Naser A. Elshimy

**Affiliations:** ^1^ Zoology Department, Faculty of Science, Assiut University, Assiut, Egypt; ^2^ Zoology Department, Faculty of Science, New Valley University, New Valley, Egypt

**Keywords:** silver nanoparticles, nitrate, *Lehmannia nyctelia*, oxidative stress, DNA

## Abstract

It is known that silver nanoparticles (Ag NPs) and AgNO_3_ have harmful effects on the surrounding organisms, which may cause damage to these organisms. Therefore, the aim of this study is to detect damage caused by Ag NPs and silver nitrate to land slugs (*Lehmannia nyctelia*). In this study, the slugs were exposed to various concentrations of Ag NPs and AgNO_3_ for 15 days. The biochemical, antioxidant, lipid peroxidation (LPO), DNA fragmentation, and histopathological endpoints were assessed after 15 days of exposure to different concentrations of Ag NPs (0.04, 0.08, 0.4, and 0.8 g/L) and silver nitrate (0.04, 0.08, 0.4, and 0.8 g/L). The results show a significant decrease in total protein, total carbohydrate, superoxide dismutase, and GST and a significant increase in total lipid, LPO, and DNA fragmentation after exposure to Ag NPs and AgNO_3_ for 15 days compared with the control group. Histopathiological alterations were observed in the digestive glands which were indicated by histochemical staining. We concluded that exposure to AgNO_3_ and Ag NPs caused oxidative stress, genetic damage and alterations in the profile of muscle proteins and histological structure in *L. nyctelia*.

## 1 Introduction

Over the past decades, the impressive progress of nanotechnology has led to the widespread use of engineered nanoparticles in a wide variety of fields. However, their release into the environment draws attention to the effects and risks to organisms (Mekkawy et al., 2019; Naguib et al., 2020). Nanoparticles can accumulate in soil through sewage sludge application, accidental spills and airborne deposition, agrochemicals, or soil remediation (Cornelis et al., 2010). In addition, soil has been shown to be a sink for many (common) pollutants; therefore, long-term effects on soil organisms are likely (Sánchez-Aguayo et al., 1987; Rodríguez et al., 2007). These representations make soil organisms a target to be taken into account.

In recent years, the production of products containing silver nanoparticles (Ag NPs) has increased dramatically, and the release of Ag NPs into the environment is probably already occurring (Blaser et al., 2008; Luoma, 2008). Ag NPs are used as an antimicrobial component in many commercial products with a wide range of applications (Mendes et al., 2015; Vance et al., 2015). For example, they find their uses in paints, cosmetics, clothing, food packaging, electrical appliances, and biomedical products (McGillicuddy et al., 2017). This widespread use of Ag NPs has raised concerns about their environmental impact (Sakka et al., 2016).

Potential release of industrial products and wastes based on Ag NPs into the environment can lead to the accumulation of Ag NPs in soil, which have impact on soil invertebrates (Schlich et al., 2013; Bicho et al., 2016), soil microbial communities (Grün et al., 2018; Parada et al., 2019), and plants (Yan and Chen, 2019). Ag NPs after entering the environment undergo physical, chemical, and biological transformations that change their physicochemical properties, which in turn affect their toxicity (Levard et al., 2012; Levard et al., 2013; Sharma et al., 2015).

The toxicity mechanism of Ag NPs is the induction oxidative stress due to the formation of reactive oxygen species (ROS) which damage components of cells (Yang et al., 2012; Patricia et al., 2017). The effect of the presence of Ag NPs in the soil environment has not been adequately studied, but the impact of Ag NPs on earthworms has been shown (Diez et al., 2015; Garcia et al., 2016; Patricia et al., 2017). Because Ag ions dissociate from Ag NPs, it is important to understand the impact of both forms of silver, which can help us in risk assessment.

Gastropods are known to be efficient mineral scavengers and respond to pollution in a sensitive and measurable manner; therefore, they are widely used as indicators of environmental pollution in soil (Viard et al., 2004; Snyman et al., 2005). Land snails and slugs have a high capacity to accumulate heavy metals and are considered important species for monitoring the bioavailability of metal constituents in soil compared to other invertebrates (Lanno and McCarty, 1997). The digestive gland is mainly where heavy metals accumulate in slugs (Marigómez et al., 2002; Johnston et al., 2010) and plays a critical role in metal detoxification (Kammenga et al., 2000). In slugs, the digestive gland consists of three different types of cells: digestive cells (the most abundant), excretory cells, and calcium (basophilic) cells (Shiroky et al., 1993).

Biomarkers such as total protein (TP), total carbohydrate (TC), total lipid (TL), superoxide dismutase (SOD), and GST enzymes, as well as lipid peroxidation (LPO) and DNA fragmentation at the suborganism level, are considered indicators of stress response (Huggett, 2018).

Several biomarkers have been used as effective tools at the cellular level due to their sensitivity, rapid response, and precise relationship between toxicant exposure and associated biological responses (Connon et al., 2012). Oxidative stress is a process caused by a steady-state imbalance between oxidants and antioxidants that plays a role in the pathogenesis of many diseases, including those caused by environmental agents, including metal ions (Samet and Wages, 2018). This can damage proteins and DNA and cause LPO (Wible and Bratton, 2018). The major toxicity of Ag NPs in environmental organisms causes ROS generation and LPO (Terevinto et al., 2010; J Cataldo R et al., 2011).

This study aimed to investigate the toxicity of different concentrations of Ag NPs on slugs compared with the same concentrations of the soluble form of the metal (AgNO_3_) using multiple biomarkers such as antioxidants and biochemical biomarkers, genotoxic damage, and histopathological changes.

## 2 Materials and Methods

### 2.1 Chemicals

Silver nitrate (AgNO_3_; with 99.8% purity and a molecular weight of 169.873 g/mol) as colorless crystals was obtained from Sigma-Aldrich (St. Louis, MO, United States).

The Ag NPs as powder with spherical-shaped particles (with 99.9% purity, a molecular weight of 107.87 g/mol, and an average particle size of <100 nm) were purchased from Sigma-Aldrich (St. Louis, MO, United States).

### 2.2 Characterization of AgNO_3_ and the Ag NPs

#### 2.2.1 TEM

NP characterization was performed using transmission electron microscopy (TEM) at TEMU, Assiut University (JEOL JEM-1200 EX II).

#### 2.2.2 UV–Vis Spectroscopy

The surface plasmons of AgNO_3_ and the Ag NPs were determined by collecting an absorption profile in the wavelength range of 250–600 nm using UV–visible (UV–Vis) spectroscopy (SHIMADZU UV-3101PC UV–Vis–NIR).

#### 2.2.3 X-ray Diffraction Patterns

The structures of AgNO_3_ and the Ag NPs were examined by X-ray diffraction (XRD). The spectra were recorded at scanning mode on PW 2103 from Philips (Netherlands). The X-ray diffractometer used Cu Kα radiation (*λ* = 1.5406 Å) in the range of 2θ = 4°–80°.

### 2.3 Stock Preparation

The stock solution (AgNO_3_ and Ag NPs) was prepared according to production protocol and stored at 4°C in the dark. The stock solution (1 g/L) was prepared using purified water (Milli-Q) and sonicated before each use. Dilutions of the experimental concentrations were prepared from this stock solution before starting each experiment.

### 2.4 Sample Collection

Specimens of *Lehmannia nyctelia* (about 180 samples) (with a weight of 1 ± 0.4 g and a length of 1.5 ± 0.5 cm) were harvested during autumn at the Assiut University Farm, Assiut Governorate, Egypt, and transported to the Ecology Laboratory at Assiut University. The slugs were kept under normal laboratory conditions, kept in plastic containers with soil from their natural habitat, and fed fresh lettuce daily for 15 days until acclimatization.

### 2.5 Experimental Design

The slugs were randomly divided into nine groups (one control group and eight groups treated). Each group consisted of 20 samples and kept in plastic containers containing a mass of soil (30 g). Each container was covered with a perforated cloth for ventilation.

The control group was fed fresh lettuce impressed in 10 ml of distilled water. On the other hand, the treated groups were fed fresh lettuce impressed in various concentrations of Ag NPs and AgNO_3_ mixed with distilled water.

The first group was the control group, and the second, third, fourth, and fifth groups were exposed to 0.04-, 0.08-, 0.4-, and 0.8-g/L Ag NPs, respectively, for 15 days. The sixth, seventh, eighth, and ninth groups were exposed to 0.04-, 0.08-, 0.4-, and 0.8-g/L AgNO_3_, respectively, for 15 days. At the end of the experiments, the surviving slugs were used for biochemical analysis, antioxidant biomarkers, and histological studies.

### 2.6 Estimation of the Silver Ions

The estimation of the Ag by element and atomic mass in the sample was done by EDX (JSM-5400LV scanning microscope) at EMU at Assiut University with a link ISIS operator’s manual.

### 2.7 Measurement of the Biochemical Parameters

The whole body of *L. nyctelia* was homogenized in 1 ml of TCA (20%), and the TP concentration was determined based on the shift observed in the absorption maximum of an acidic solution of Coomassie brilliant blue G-250 from 465 to 595 nm when protein binding occurs in accordance with the method by (Bradford, 1976). The determination of TC was performed on an aliquot (100 μL) in accordance with the method by (Moreau et al., 1984). This method uses anthrone as the reagent and glucose as the standard. The absorbance was evaluated on a spectrophotometer at a wavelength of 620 nm. The TL of the whole body of *L. nyctelia* was measured according to the method of (Goldsworthy et al., 1972).

### 2.8 Antioxidant Biomarkers

SOD was measured based on its ability to inhibit the phenazine-methosulfate-mediated reduction of nitro blue tetrazolium dye to form a red product (Nishikimi et al., 1972).

GST was determined by the spectrophotometric assay of (Ålin et al., 1985). This method measures the total GST at an absorbance of 340 nm.

### 2.9 LPO and DNA Fragmentation

LPO was measured according to the method by (Ohkawa et al., 1979) at an absorbance of 535 nm. The LPO rate was expressed in nanomoles of thiobarbituric acid reactive substance formed per hour per milligram of protein using a molar extinction coefficient of 1.56 M/cm. DNA fragmentation was measured according to the method by (Kurita-Ochiai et al., 1999) using a spectrophotometer at 575 or 600 nm against a reagent blank. The percentage of fragmented DNA was estimated using the following formula: % of fragmented DNA = fragmented DNA/(fragmented + intact DNA) × 100.

### 2.10 Histochemical Change

At the end of the experiments, the whole bodies of the slugs were immediately fixed in 70% alcohol. The fixed slugs were processed through a paraffin embedding technique. The embedded tissues were sectioned at 5–7 µm in thickness and then stained with Sudan Black, periodic acid–Schiff (PAS) and hematoxylin stain, and toluidine blue. The sectioned digestive gland was examined using an Olympus microscope (Olympus Optical Co., Ltd., Japan).

### 2.11 Statistical Analysis

The mean and standard error were analyzed using the SPSS package (SPSS 1998) at the 0.05 significance level. Data were tested for normality (Shapiro–Wilk test) and then tested for homogeneity of variances using one-way analysis of variance.

### 2.12 Ethical Statement

All experiments were carried out in accordance with Egyptian laws and university guidelines for the care of experimental animals.

## 3 Results

### 3.1 Characterization of AgNO_3_ and Ag NPs

#### 3.1.1 TEM

**FIGURE 1:**
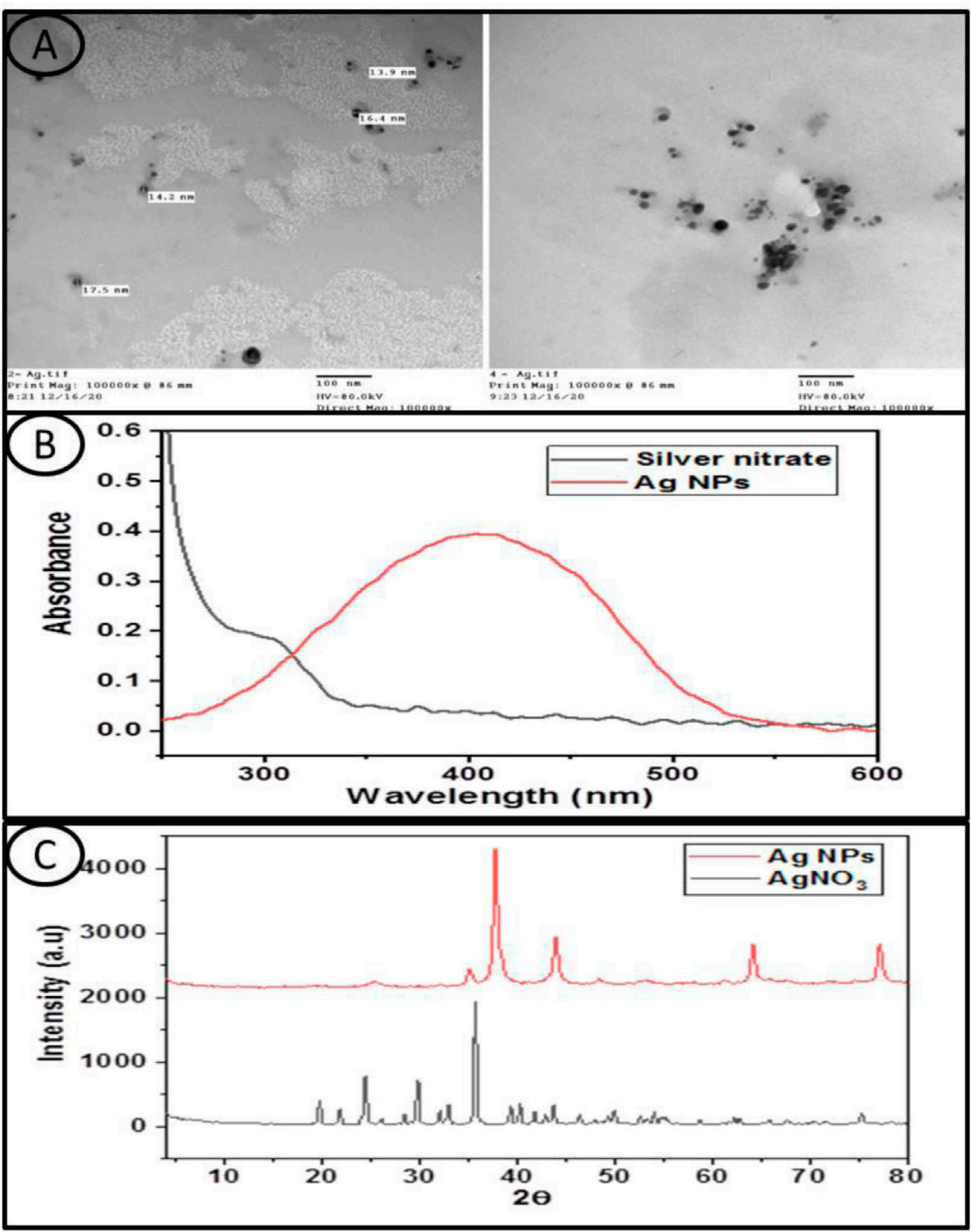
**(A)** Transmission electron microscope images of the investigated Silver Nanoparticles, **(B)** shows the UV-Vis absorption spectra of the silver nitrate and Ag NPs aqueous solution and **(C)** displayed the XRD patterns of silver nitrate and the Ag NPs used in this study.

The TEM images showed that the Ag NPs were spherical in shape with small size (Figure 1A).

#### 3.1.2 UV–Vis Spectroscopy

The spectrum of silver nitrate has no absorption characteristic peak, whereas the spectrum of the Ag NPs showed an absorption peak centered at about 405 nm (Figure 1B).

#### 3.1.3 XRD Patterns

The XRD pattern of silver nitrate exhibited several diffraction peaks, where the main higher peaks appeared at 2θ values of 35.7°, 29.8°, 24.5°, 21.8°, and 19.7°. On the other hand, the XRD pattern of the Ag NPs showed four peaks observed at 2θ values of 38°, 44°, 64.2°, and 77.1°, which can be attributed to the crystal planes (1 1 1), (2 0 0), (2 2 0), and (3 1 1), respectively, which have face-centered cubic silver crystal structures, and these values were similar with those of the standard card (JCPDS No: 04-0783) (Figure 1C).

### 3.2 Percentage of Silver

**FIGURE 2:**
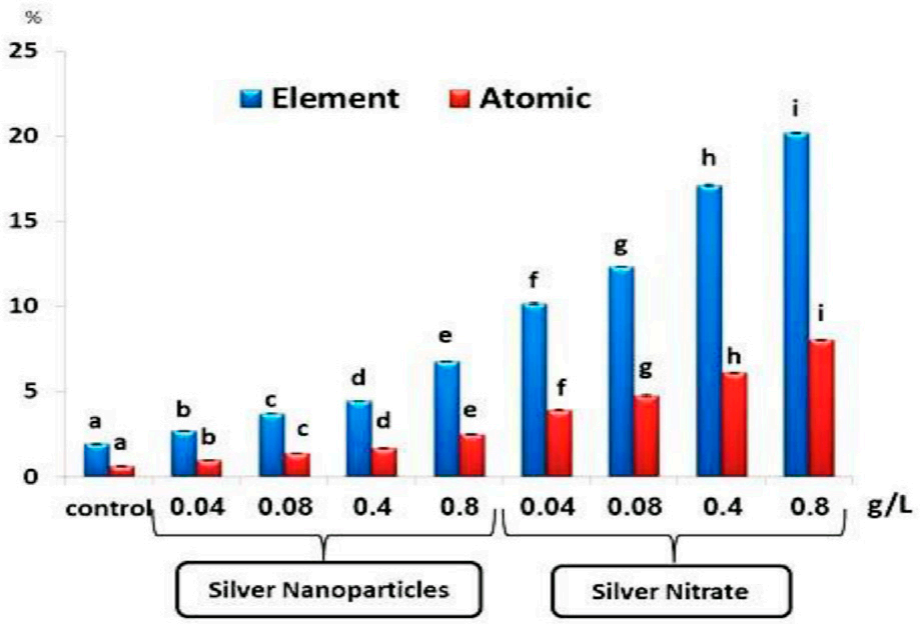
The percentage of silver as element and atomic in the total body of the slugs, (*Lehmannia nyctelia*), after 15 days of exposure. Bars represent the mean ± SE of the control and experimental groups. Different letters indicate significant differences amongst treatments (*p* < 0.05).

The percentage of Ag by element and atomic mass after exposure to different concentrations of Ag NPs (0.04, 0.08, 0.4, and 0.8 g/L) and silver nitrate (0.04, 0.08, 0.4, and 0.8 g/L) for 15 days showed a significant (*p* < 0.05) increase when compared to the control group (Figure 2).

### 3.3 Biochemical Parameters

**FIGURE 3:**
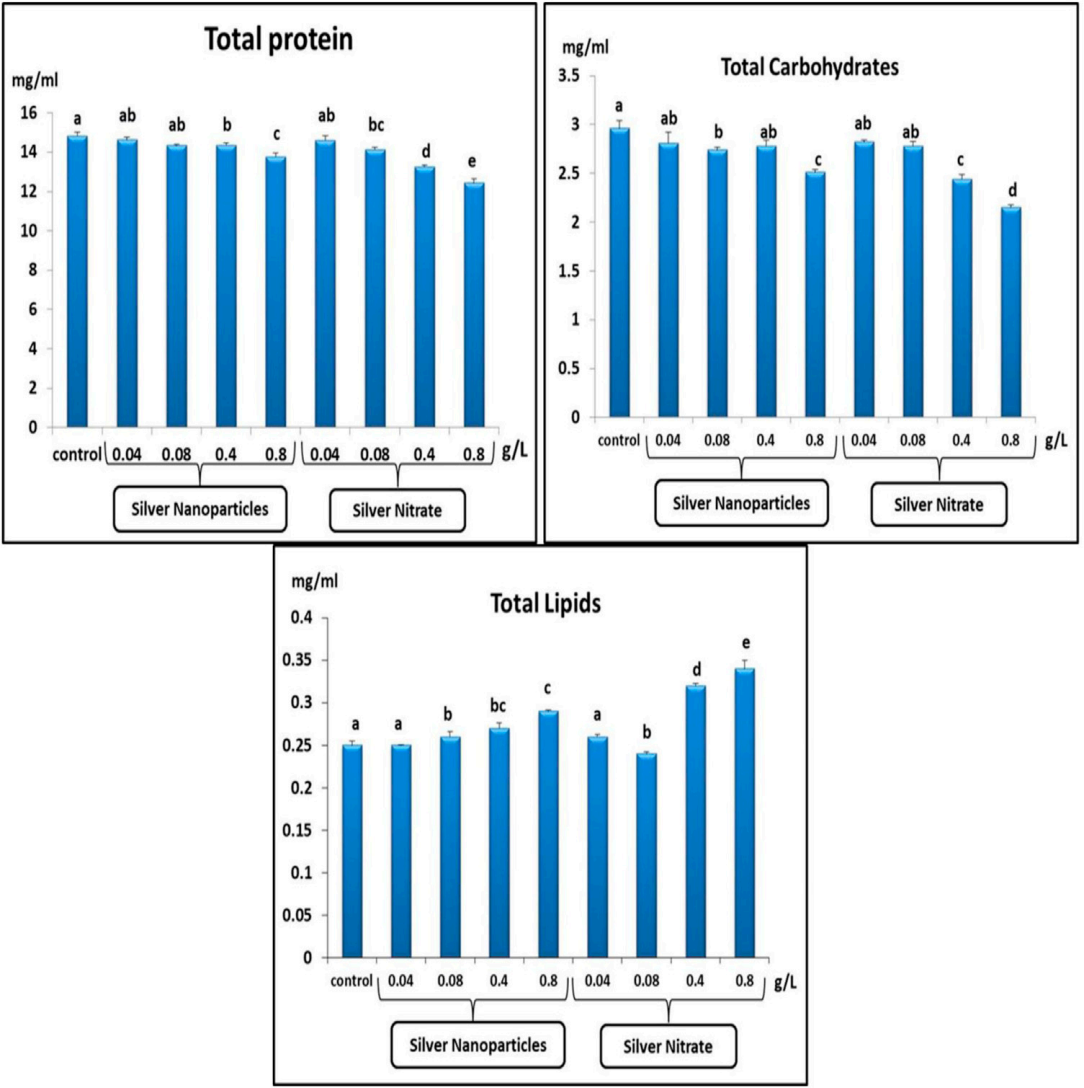
Total protein (mg/ml), total carbohydrates (mg/ml) and total lipids (mg/ml) in slugs (*Lehmannia nyctelia*) after silver nanoparticles and silver nitrite exposure for 15 days. Bars represent the mean ± SE of the control and experimental groups. Different letters indicate significant differences amongst treatments (*p* < 0.05).

The TP and TC after exposure to 0.04, 0.08, 0.4, and 0.8 g/L of Ag NPs and 0.04, 0.08, 0.4, and 0.8 g/L of silver nitrate for 15 days showed a significant (*p* < 0.05) decrease when compared to the control group (Figure 3). On the other hand, the TL showed a significant (*p* < 0.05) increase after exposure to 0.04, 0.08, 0.4, and 0.8 g/L of Ag NPs and 0.04, 0.08, 0.4, and 0.8 g/L of silver nitrate for 15 days when compared to the control group (Figure 3).

### 3.4 Antioxidant Enzymes

The SOD and GST activities in the slugs showed a significant decrease (*p* < 0.05) after exposure to 0.04, 0.08, 0.4, and 0.8 g/L of Ag NPs and 0.04, 0.08, 0.4, and 0.8 g/L of silver nitrate for 15 days in comparison with the control group (Table 1).

**TABLE 1:** Effects of silver nanoparticles and silver nitrite on the antioxidants parameters of the slugs, (*Lehmannia nyctelia*), after 15 days of exposure. Data are presented as mean ± SE. Values in the same row with different superscript letter are significantly different (*p* < 0.05). Superoxide dismutase (SOD), Glutathione -S-transfer (GST), Lipid peroxidation (LOP) and DNA fragmentation.

Treatment Parameters	Control	Silver Nanoparticals				Silver Nitrite			
		0.04 g/L	0.08 g/L	0.4 g/L	0.8 g/L	0.04 g/L	0.08 g/L	0.4 g/L	0.8 g/L
GST (mg/gm)	0.63 ± 0.009^a^	0.61 ± 0.001^ab^	0.61 ± 0.001^ab^	0.55 ± 0.006^b^	0.53 ± 0.006^c^	0.60 ± 0.003^a^	0.59 ± 0.003^b^	0.48 ± 0.01^d^	0.42 ± 0.003^e^
SOD (U/gm)	2.53 ± 0.04^a^	2.43 ± 0.05^a^	2.28 ± 0.03^b^	2.19 ± 0.02^bc^	2.08 ± 0.03^c^	2.44 ± 0.05^a^	2.16 ± 0.03^bc^	2.07 ± 0.01^c^	1.84 ± 0.03^d^
DNA fragmentation (%)	10.03 ± 0.4^a^	11.58 ± 0.15^b^	12.5 ± 0.19^c^	14.49 ± 0.18^d^	16.38 ± 0.15^e^	11.9 ± 0.13^b^	12.8 ± 0.12^c^	15.01 ± 0.18^d^	17.5 ± 0.15^e^
LOP (nmol/ml)	0.11 ± 0.02^a^	0.19 ± 0.03^b^	0.22 ± 0.01^c^	0.28 ± 0.02^d^	0.3 ± 0.02^e^	0.19 ± 0.02^b^	0.25 ± 0.04^c^	0.29 ± 0.02^d^	0.32 ± 0.04^e^

### 3.5 DNA Fragmentation and LPO

LPO and DNA fragmentation after exposure to 0.04, 0.08, 0.4, and 0.8 g/L of Ag NPs and 0.04, 0.08, 0.4, and 0.8 g/L of silver nitrate for 15 days showed a significant increase (*p* < 0.05) in comparison with the control group (Table 1).

### 3.6 Histochemical Change

#### 3.6.1 PAS and Hematoxylin Reaction for Carbohydrates in the Digestive Gland of the Slugs (*L. nyctelia*)

##### 3.6.1.1 Control Group

**FIGURE 4:**
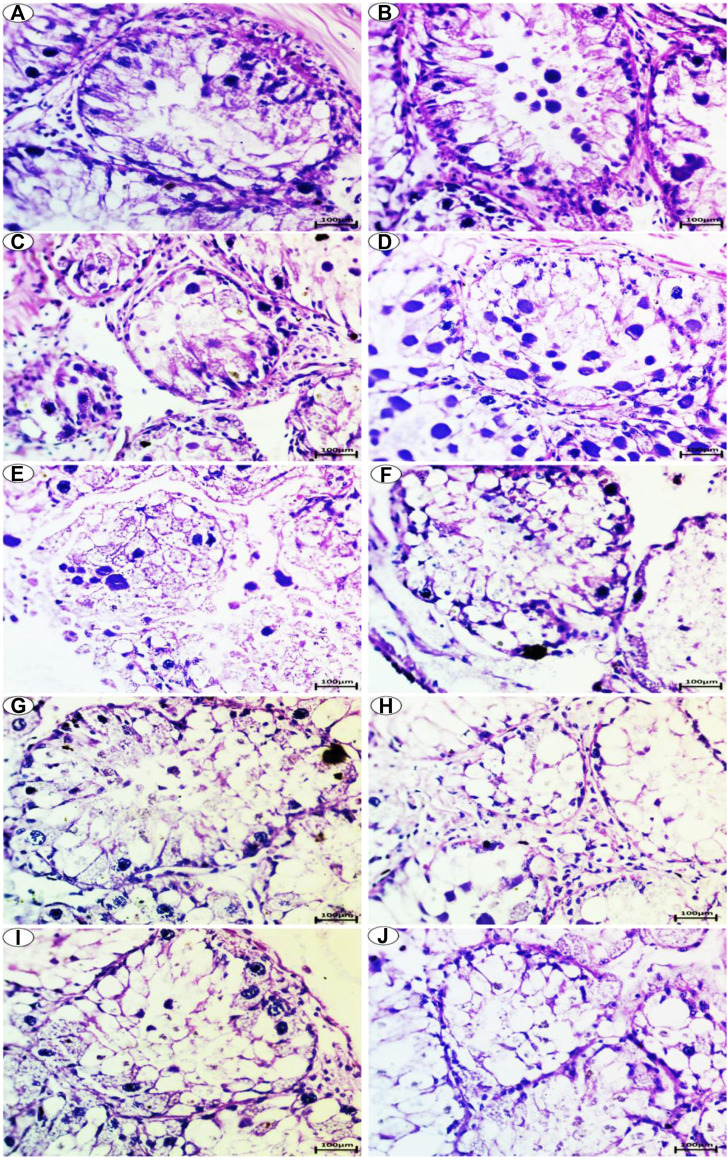
Photomicrograph of digestive gland of Slug *Lehmannia nyctelia* after the exposure to **(A,B)** Control group, **(C)**; 0.04 g/L of Ag NPs, **(D)**; 0.08 g/L of Ag NPs, **(E)**; 0.4 g/L of Ag NPs and **(F)**; 0.8 g/L of Ag NPs, **(G)**; 0.04 g/L of silver nitrate, **(H)**; 0.08 g/L of silver nitrate, **(I)**; 0.4 g/L of silver nitrate, **(J)**; 0.8 g/L of silver nitrate. (PAS and hematoxylin, scale bar 100 μm).

PAS staining for carbohydrates in the digestive gland showed a positive reaction of polysaccharides and a high amount of carbohydrates in the control group, as shown in Figures 4A,B.

##### 3.6.1.2 Treatment Exposure to Ag NPs

In the group treated with 0.04-g/L Ag NPs, a slight decrease in the amount of carbohydrates was observed, as shown in Figure 4C. In the group treated with 0.08-g/L Ag NPs, a moderate amount of carbohydrates was observed, as shown in Figure 4D.

The group exposed to 0.4-g/L Ag NPs showed depletion in carbohydrates, as shown in Figure 4E, whereas the group exposed to 0.8-g/L Ag NPs showed more depletion in the amount of carbohydrates, as shown in Figure 4F.

##### 3.6.1.3 Treatment Exposure to Silver Nitrate

The group treated with 0.04-g/L silver nitrate showed a moderate amount of carbohydrates, as shown in Figure 4G, whereas the group exposed to 0.08-g/L silver nitrate showed depletion in the amount of carbohydrates, as shown in Figure 4H.

The group exposed to 0.4-g/L silver nitrate showed more depletion in the amount of carbohydrates, as shown in Figure 4I, whereas the group exposed to 0.8-g/L silver nitrate showed greater depletion in the amount of carbohydrates, as shown in Figure 4J.

#### 3.6.2 Toluidine Blue for Proteins or Mucopolysaccharide Contents in the Digestive Gland of the Slugs (*L. nyctelia*)

Toluidine blue staining (pH of 2.5) was performed for acidic structures in the nucleus and cytoplasm and for colored structures with the same color of the dye termed as orthochromatic structures.

##### 3.6.2.1 Control Group

**FIGURE 5:**
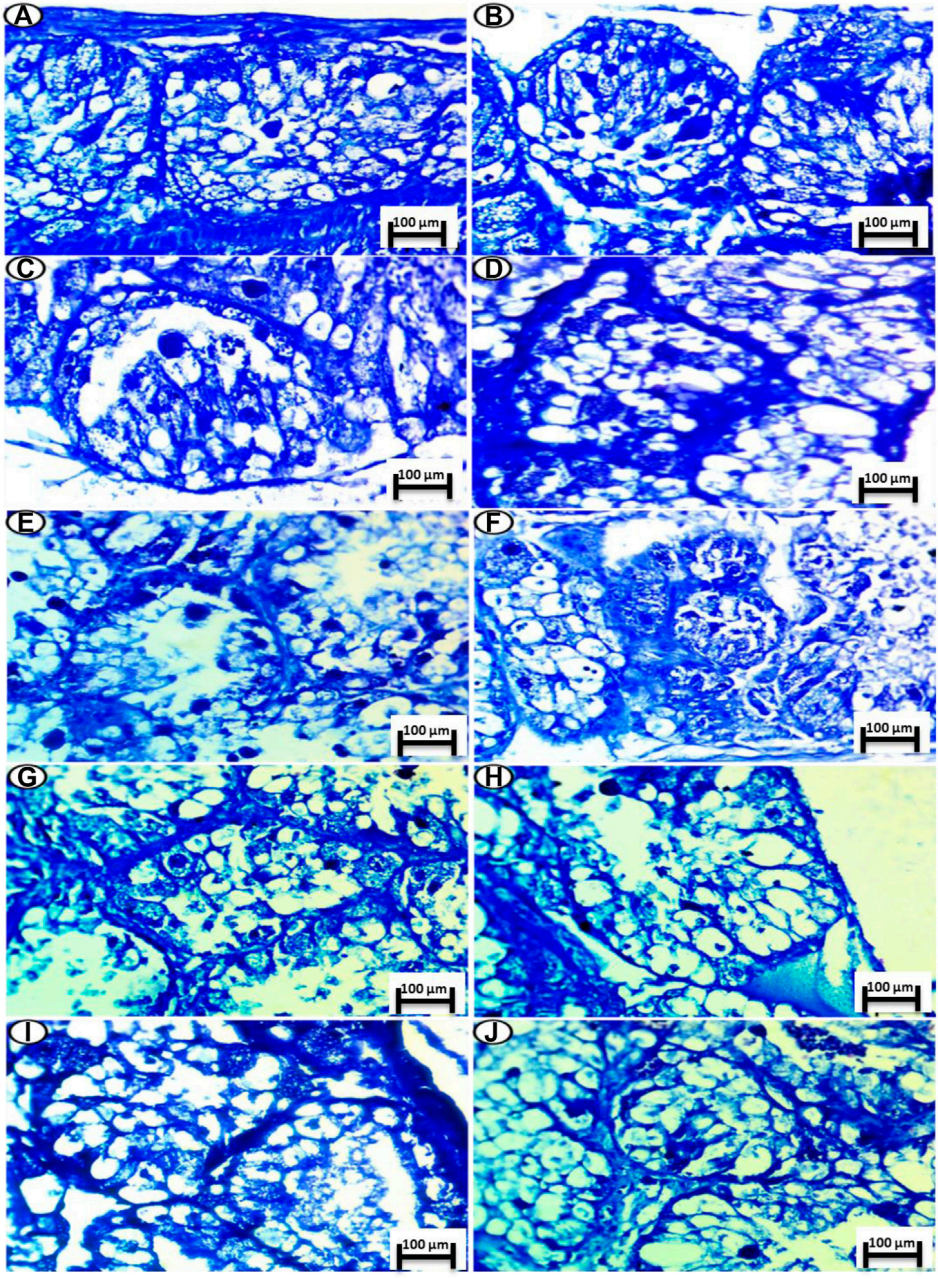
Photomicrograph of digestive gland of Slug *Lehmannia nyctelia* after the exposure to **(A,B)** Control group, **(C)**; 0.04 g/L of Ag NPs, **(D)**; 0.08 g/L of Ag NPs, **(E)**; 0.4 g/L of Ag NPs and **(F)**; 0.8 g/L of Ag NPs, **(G)**; 0.04 g/L of silver nitrate, **(H)**; 0.08 g/L of silver nitrate, **(I)**; 0.4 g/L of silver nitrate, **(J)**; 0.8 g/L of silver nitrate. (Toluidine blue, scale bar 100 μm.

The control group showed a normal intensity of blue color for localization of mucopolysaccharide contents (orthochromatic structures) in the cells of both the nuclei and cytoplasm and the digestive gland basement membrane and boundary of lining cells, as shown in Figures 5A,B.

##### 3.6.2.2 Treatment Exposure to Ag NPs

The group exposed to 0.04 g/L of Ag NPs showed a slight decline in the intensity of staining in comparison with the control group (Figure 5C), while this intensity was decreased again in the group exposed to 0.08 g/L of Ag NPs and with more deposition at its basement membrane (Figure 5D).

The groups exposed to 0.4 and 0.8 g/L of Ag NPs showed severe depletion of mucopolysaccharide contents, and this shortage was noticed in the last groups. In addition, the main deepest coloration was noticed in the nuclei and faint reactions were noticed in the cytoplasm (Figures 5E,F).

##### 3.6.2.3 Treatment Exposure to Silver Nitrate

In the silver-nitrate-treated groups, there is amelioration in the color deposition of the mucopolysaccharide contents especially in the basement membrane and the fine granules inside the lining cells, and this decrement was noticed for concentrations of 0.04 and 0.08 g/L of silver nitrate (Figures 5G,H), whereas a slight decline was noticed for concentrations of 0.4 and 0.8 g/L of silver nitrate (Figures 5I, J).

#### 3.6.3 Sudan Black for Conjugated Lipids in the Digestive Gland of the Slugs (*L. nyctelia*)

**FIGURE 6:**
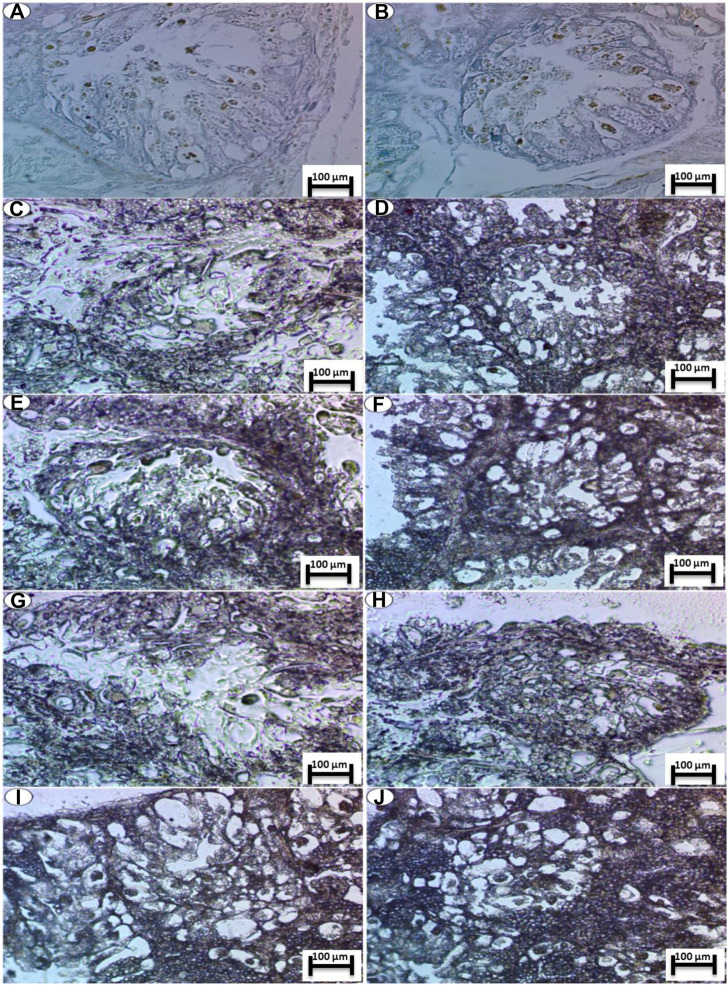
Photomicrograph of digestive gland of Slug *Lehmannia nyctelia* after the exposure to **(A,B)** Control group, **(C)**; 0.04 g/L of Ag NPs, **(D)**; 0.08 g/L of Ag NPs, **(E)**; 0.4 g/L of Ag NPs and **(F)**; 0.8 g/L of Ag NPs, **(G)**; 0.04 g/L of silver nitrate, **(H)**; 0.08 g/L of silver nitrate, **(I)**; 0.4 g/L of silver nitrate, **(J)**; 0.8 g/L of silver nitrate. (Sudan black, scale bar 100 μm).

The Sudan Black reaction showed a moderate accumulation of lipids in the digestive gland of the slugs (*L. nyctelia*) in the control group, whereas a large amount of lipids (Figure 6) was accumulated and observed in the digestive gland for the Ag NP- and silver-nitrate-treated groups.

## 4 Discussion

Little is known about the negative effects of AgNO_3_ and Ag NPs on *L. nyctelia* in soil, while many industries are currently using Ag NPs, and there is a possibility of increased environmental and human exposure with harmful toxicological consequences (Martirosyan et al., 2014). The characterization of AgNO_3_ and Ag NPs explains many properties of the materials used in the experiments, and the result of UV–Vis spectroscopy and the XRD patterns indicate that the Ag NPs have a spherical shape with a small size and a regular distribution, which is a characteristic of the surface plasmon resonance of silver in accordance with that by (Vu et al., 2018).

The slugs died during the experiments at a high rate with the AgNO_3_ treatments but at a low rate with the Ag NP treatments, indicating that AgNO_3_ does exhibit very acutely toxicity to *L. nyctelia* within 15 days, whereas the Ag NPs does exhibit acutely toxicity to *L. nyctelia* within 15 days. Many studies have shown that much of the toxicity of heavy metals is closely related to the production of ROS in biological systems (Valko et al., 2005). Oxidative stress determines the ability of ROS to damage cellular components such as cell membranes, proteins, DNA, and RNA (Radwan et al., 2010). In mollusks, heavy metals can cause a state of general stress, resulting in a decrease in their ability to adapt to hypoxia.

Proteins are involved in the structure of the cell. In chronic stress, proteins become a source of energy (Radwan et al., 2008). The results showed a significant reduction in the TP content. Similarly (Laila and Genena, 2011), found that the level of TP in *Monacha cantiana* and *Eobania vermiculata* decreased after exposure to molluscicides, while (Radwan and Mohamed, 2013) observed a significant increase in the protein rate in snails treated with 0.6 LD of imidacloprid. The accumulation of heavy metals in mollusks is often associated with a decrease in proteins belonging to metallothioneins, which play an important role in metal homeostasis.

Carbohydrates are the main and immediate source of energy. When oxidative stress occurs, carbohydrate stores are depleted to meet the increased need for energy (Arasta et al., 1996). Our results show a significant decrease in carbohydrate rates. The study by (Radwan et al., 2008) indicated a significant decrease in carbohydrate rate in *E. vermiculata* after exposure to pesticides. Unlike in the work by (Barky et al., 2012), atrazine caused a toxic effect by increasing the glucose concentration in snails (*Biomphalaria alexandrina*).

Under adverse conditions, slugs needed the energy to eliminate toxicants and stress. Slugs have a limited amount of carbohydrates, and proteins are the next alternative source of energy to meet the increased energy requirements (Radwan et al., 2008). (Padmaja and Balapara 1994) suggested that the reduction in protein content in the tissues of freshwater snails (*Bellamya dissimilis*) after exposure to pesticides may be due to some mechanisms: 1) the formation of lipoproteins, which are used to repair damaged cells and tissues, and 2) direct use by cells to meet energy needs (Radwan et al., 2008). Lipids are also a source of energy and important for the formation of cells and tissues (Araghi and Bastami, 2011). In our results, the TLs were significantly increased, which is consistent with the results by (El-Shenawy et al., 2012), which showed a significant increase in TLs in *E. vermiculata* collected from contaminated areas. Also, these results are consistent with the results by (Laila and Genena, 2011); they found that the treatment with all tested molluscicides for 3 days increased the TL levels in *E. vermiculata*.

SOD plays an important role in the protection against free radicals and oxidative stress (Menvielle-Bourg, 2005; Roubalová et al., 2015). SOD inhibition under the influence of NPs is one of the most important biomarkers of oxidative stress. In our study, a decrease in SOD activity was observed, as in the results by (Mwaanga et al., 2014), which recorded a decrease in SOD after exposure to Cu, CuO, and ZnO NPs on earthworms in soil. As noted by researchers elsewhere (Gill and Tuteja, 2010), GST plays an important role as an antioxidant, maintaining the integrity of proteins and enzymes. It is known that the restoration of GST in antioxidant processes is carried out due to several other processes (Lushchak, 2012). Thus, in this study, GST depletion could be caused by several processes (Gomes et al., 2015). showed that exposure of *Eisenia fetida* to Ag^+^ mixed with OECD soil at 25–200 mg/kg for 28 days resulted in a decrease in CAT and GST activities. Inhibition of GST activity can occur both due to the direct action of the metal on the enzyme and indirectly through the production of ROS that directly interacts with the enzyme and the depletion of its GSH (Roling and Baldwin, 2006). This explanation may be the reason for the decrease in GST activity that was induced in the present study in the case of slugs exposed to Ag NPs and silver nitrate.

Two mechanisms of Ag NP action have been proposed: 1) the release of Ag^+^ ions during long-term incubation in soil and the induction of ROS (McShan et al., 2014) and 2) the direct induction of ROS (Wijnhoven et al., 2009). Both mechanisms can have negative effects on oxidative stress and genotoxicity associated with exposure to Ag NPs, which can damage lipids, proteins, and nucleic acids and stimulate SOD, CAT, and GST production (McShan et al., 2014; Durán et al., 2016; Wang et al., 2017). Ag NPs can also cause toxicity through surface oxidation, which results in the release of Ag^+^, which can interact with lipid molecules, nucleic acids, and proteins in biological systems (McShan et al., 2014).

LPO has been studied in programs of environmental monitoring. Increased LPO has been reported in different snails exposed to laboratory or environmental pollutants (Al-Daihan et al., 2010; Barky et al., 2012; El-Shenawy et al., 2012). Mendes et al. (2015) showed in a similar study when *Folsomia candida* was exposed to Ag^+^ and Ag NPs and found that the increase in LPO was more pronounced in those exposed to Ag^+^ than those exposed to Ag NPs. This result is consistent with previous results that showed that copper exposure induces oxidative stress in snail tissues, as evidenced by an increase in LPO (Viarengo et al., 1990).

Genotoxicity is one of the most important chemical toxicity tests and risk assessments; however, little is known about the genotoxicity of AgNO_3_ and Ag NPs (Sayed, 2016; Sayed and Soliman, 2017; Sayed and Younes, 2017; Mahmoud et al., 2019; Mekkawy et al., 2019; Naguib et al., 2020), especially for terrestrial organisms *L. nyctelia*. It is known that ROS reacts with the DNA molecule, causing damage to purine and pyrimidine bases, as well as to the DNA backbone (Ali et al., 2015).

The comparison between silver nitrate and Ag NPs with the same concentrations showed that the antioxidant enzyme activity in the slugs exposed to silver nitrate was higher than that in the slugs exposed to the Ag NPs. There are many reasons why silver nitrate may be more toxic than Ag NPs: 1) The solubility of silver nitrate is much higher than the solubility of Ag NPs; 2) in the molar ratio, the concentration of silver nitrate ions is higher than the concentration of Ag NPs of equal mass; and 3) the elimination rate of Ag NPs is higher than that of silver nitrate (Ribeiro et al., 2015).

Histochemical examination of the digestive gland of *L. nyctelia* treated with AgNO_3_ and Ag NPs highlighted important qualitative changes because of the different concentrations tested. Indeed, nanoparticle exposure can cause very significant cytological alterations in the digestive gland, which plays a crucial role in the detoxification of pollutants (Frías-Espericueta et al., 2008). The histochemical changes are expected to be useful biomarkers of AgNO_3_ and Ag NP exposure (Snyman et al., 2009). The histopathological responses of the several organs (digestive gland) of *L. nyctelia* exposed to increasing concentrations of AgNO_3_ and Ag NPs in food occurring through reactions involving epithelial hyperplasia at the lowest doses tested the cell destruction and are accompanied by a proliferation of connective tissues and necrosis at high concentrations.

The results of this study can demonstrate the environmental consequences of the release of AgNO_3_ and Ag NPs into ecosystems. These biomarkers have also opened up broad prospects in essential toxicology as *L. nyctelia* is exposed to environmental pollutants. Our results provide regulators and industries with important information to determine whether AgNO_3_ and Ag NPs need to be monitored and regulated.

## 5 Conclusion

In conclusion, exposure to AgNO_3_ and Ag NPs caused changes in the antioxidant biomarkers (SOD and GST), DNA damage, LPO, and changes in the profile of muscle proteins with changes in the histopathological reactions of *L. nyctelia*, which can be considered as the cause of death of *L. nyctelia*.

## Data Availability

The original contributions presented in the study are included in the article/supplementary materials, further inquiries can be directed to the corresponding author.
